# Fidgetin as a potential prognostic biomarker for hepatocellular carcinoma

**DOI:** 10.7150/ijms.49913

**Published:** 2020-10-16

**Authors:** Bin Zhou, Jisheng Wang, Jing Gao, Junqing Xie, Yiming Chen

**Affiliations:** Department of Hepatobiliary Surgery, The Second Affiliated Hospital and Yuying Children's Hospital of Wenzhou Medical University, Wenzhou 325000, Zhejiang, China.

**Keywords:** hepatocellular carcinoma, Fidgetin (FIGN), biomarker, progression, prognosis

## Abstract

**Background:** Fidgetin (FIGN), a conserved ATP-dependent enzyme, is regarded as a hepatocellular carcinoma (HCC) risk gene, but the prognostic implication of FIGN in HCC remains obscure. In this study, we investigate the expression of FIGN in HCC and to evaluate its prognostic value.

**Methods:** A total of 216 patients with HCC who experienced hepatectomy were recruited in this study. The expression of FIGN in HCC samples was evaluated by quantitative real-time PCR, immunohistochemistry and immunoblotting analysis. And Cox regression model was used to evaluate the prognostic value of all covariates.

**Results:** Of the 216 HCC patients, 67 (31.0%) had tumors with high FIGN expression and 149 (69.0%) had tumors with low FIGN expression. FIGN expression was positively correlated with TNM stage (*P* = 0.039), tumor with incomplete capsule (*P* = 0.036), microvascular invasion (*P* = 0.023), and portal vein tumor thrombus (*P* = 0.003). High expression of FIGN indicated shorter overall survival (OS) (hazard ratio: 4.569, *P* = 0.036) and disease-free survival (DFS) (hazard ratio: 6.487, *P* = 0.001).

**Conclusion:** Our results indicate that high Fidgetin expression is associated with tumor progression and suggest a worse prognosis in HCC. Fidgetin might serve as a potential target for therapy.

## Introduction

Each year an estimated 745,000 people worldwide die from liver cancer, making it the fifth most common malignancy and the second largest contributor to cancer related death 1, 2. Hepatocellular carcinoma (HCC), a tumor of the parenchymal cells, accounts for approximately 80% of cases 1. Hepatoma metastasis is primarily responsible for the poor prognosis of patients with HCC and is the major obstacle to improving prognosis. Extensive intrahepatic invasion and extrahepatic metastasis are the leading reasons of deaths in patients with HCC 3. Thus, to identify effective biomarker that predicts cancer metastasis may improve outcome and reduce the mortality of HCC.

Cellular proliferation and migration are critical steps in the tumor progressing process. A series of molecular changes could enable tumor cells to be more aggressive by altering motile properties. Microtubules (MT) engage in cell cycle, trafficking, signaling and migration 4, while MT inhibitors block the tumor angiogenesis by reducing the MT polymerization and subsequently lead to tumor regression 5. Fidgetin (FIGN), a conserved ATP-dependent enzyme, plays an important role in MT-severing capability. Functions of FIGN have been indicated in DNA synthesis, mitosis, meiosis, and cellular migration by acting tyrosinated MT at the leading edge 6-8. Recently, a publication has showed that FIGN is elevated in both murine and human hepatic tumor tissues; and overexpression of Fign can promote mouse hepatocyte invasion through extracellular matrix 9. Therefore, FIGN may play an important role in tumor progression by regulating hepatoma metastasis. However, whether FIGN has an association with prognosis in HCC remains unclear.

In this study, we investigated the gene and protein levels of FIGN in human HCC tissues, and analyzed its correlation with the prognosis for HCC patients.

## Methods

### Patients and tissue samples

A total of 216 patients with HCC who experienced hepatectomy at the Department of Surgery in The Second Affiliated Hospital and Yuying Children's Hospital of Wenzhou Medical University between January 2012 and December 2015 were included in this study. Neoadjuvant chemotherapy or radiotherapy was not carried out before surgery in any of the cases. Fresh samples from pathologically representative tumor regions and paired adjacent normal hepatic tissues were obtained. Patients' tumor characteristics including tumor diameter, tumor envelope, number of tumors, satellite nodule, microvascular invasion (MVI) and portal vein tumor thrombus (PVTT) were summarized. Other clinicopathological data such as gender, age, hepatitis B virus (HBV) infection and survival data were obtained from medical records. Clinical stages of all HCC patients were determined according to Barcelona Clinic Liver Cancer (BCLC) staging system and the 7^th^ edition of American Joint Committee on Cancer TNM staging system 10, 11. Overall survival (OS) was defined as the duration from the initiation of hepatectomy to the date of death or the last follow-up, whichever occurred first. Disease-free survival (DFS) was defined as the time from the date of hepatectomy to the date of disease recurrence or the last follow-up. The study protocol was reviewed and approved by the Institutional Research Ethics Committee of the Second Affiliated Hospital of Wenzhou Medical University. Written consents were obtained from patients prior to commencing the study.

### Immunohistochemistry (IHC)

Tissue sections were prepared from the formalin-fixed paraffin embedded specimens. Antigen unmasking was performed with 10 mM citrate buffer (pH 6) at 99 °C for 10 min. Endogenous peroxidase was quenched with a solution of methanol with 3% H_2_O_2_, and 3% bovine serum albumin was used to block nonspecific staining. Slides were incubated with rabbit anti-FIGN (1:50) overnight at 4 °C. Chromogenic staining was performed with a peroxidase conjugated secondary antibody for 30 min and DAB reagents (Dako, Carpinteria, CA, USA) for 5 min at room temperature. The density of positive cells was analyzed by Image-Pro Plus 6.0 (Meida Cybernetics, Inc., Bethesda, MD, USA).

Each section was calculated an intensity value for hepatocyte FIGN staining by two experienced pathologists in a blinded manner. A histoscore (H-score) based on the percentage of immunoreactive cells and the intensity of immunostaining was divided into four categories (negative, 0; weak: 1; moderate: 2; strong: 3 ). Negative: essentially absent staining; 1: faint, discontinuous staining; 2: reduced staining; 3: strong staining. Subsequently, for statistical analyses, the negative or weakly positive cases were classified as the low expression group, whereas the moderately or strongly positive cases were classified as the high expression group.

### Reverse transcription and quantitative real-time PCR (qRT-PCR)

Total RNA was extracted from the pathologically representative tumor regions and matched adjacent healthy hepatic tissues of 24 patients by TRIzol^®^ Reagent (Invitrogen Technologies, Carlsbad, CA, USA) according to the manufacturer's instruction. Single-stranded cDNA was synthesized using M-MLV reverse transcriptase (Invitrogen Technologies) from total RNA. Oligo (dT) 18 was used as the RT primers for reverse transcription of mRNAs. In the qRT-PCR, each sample was run in triplicate in a 10 μl reaction with 250 nM forward and reverse primers, 5 μl of SYBR Green Supermix (Bio-Rad, Berkeley, CA, USA) and 10 ng of cDNA. *GAPDH* was used as control. Reactions were performed in the BIO-RAD CFX Real-Time System and relative mRNA expression levels were calculated using the ΔCt method (2^-ΔΔCt^). The primers were as follows: *FIGN*, forward 5'-GTCGCCAAGTGGTTAGGAGAAGC-3' and reverse 5'-CTCGCTGTGCTGTCAGGAAGTG-3'; *GAPDH*, forward 5'-GGAAGGTGAAGGTCGGAGT-3' and reverse 5'-CCTGGAAGATGGTGATGGG-3'.

### Western blot (WB)

Liver tissues were lysed with 0.5% NP40 buffer containing protease inhibitor cocktail on ice, and the supernatants were collected by centrifugation at 13,000 rpm at 4 °C for 15 min. Protein samples were separated by 10% SDS-PAGE and transferred to nitrocellulose membranes. Membranes were blocked with 5% skimmed milk and then incubated with primary antibody overnight at 4 °C. The dilutions of rabbit anti-FIGN (Abcam, Cambridge, UK) were 1:500. The line densitometry was quantified by Image J software (version 1.61, National Institutes of Health, Bethesda, MD, USA).

### Follow-up strategy

Follow-up time was calculated from the time of the operation to the day of last follow-up or death. All patients were followed up every 2-3 months in the first 2 years and then evaluated every 3-5 months. Follow-up was until May 2019.

### Statistical analyses

Statistical analysis was undertaken using SPSS version 19.0 (SPSS Inc., Chicago, IL, USA). For each variable, Kolmogorov-Smirnov normality was performed. A Kruskal-Wallis test was applied for the non-normally distributed variables. To evaluate relationships between immunohistochemical results and clinicopathological characteristics, Chi-square test was used for categorical variables. OS and DFS were assessed by the Kaplan-Meier method and the log-rank test was used to estimate the differences. Multivariate analysis of prognostic factors was performed by Cox proportional hazard model. The significance level for statistical testing was set at *P* < 0.05.

## Results

### Patient characteristics

We analyzed 216 HCC patients, comprising 141 males (65.3%) and 75 females (34.7%). The median age of these patients at operation was 51 years (range from 29 to 81 years). 139 (64.4%) patients had HBV infection. 113 tumors (52.3%) were smaller than 5 cm, whereas, 103 tumors (47.7 %) had a minimum diameter of 5 cm or more. There were 127 cases (58.8%) with intact capsule and 89 cases (41.2%) with incomplete envelope. 177 cases (81.9%) had solitary tumor, and 39 cases (18.1%) had multiple tumors. In terms of MVI, 63 cases (29.2%) were positive and 153 cases (70.8%) were negative. There were 43 cases (19.9%) had satellite nodule and 36 cases (16.7%) had PVTT at initial operation. 148 (68.5%) and 68 (31.5%) cases were classified as stage I and stage II/III according to the TNM staging system, respectively. And 143 cases (66.2%) were classified as stage A and 73 cases (33.8%) as stage B/C according to BCLC staging system. The median time point of follow-up was 47.3 months (ranging from 0.24-76.0 months). 161 patients (74.5%) died from cancer-related issues. The clinicopathological characteristics of the entire cohort are presented in **Table 1.**

### Relationship between FIGN expression and clinicopathological characteristics

**Figure 1:**
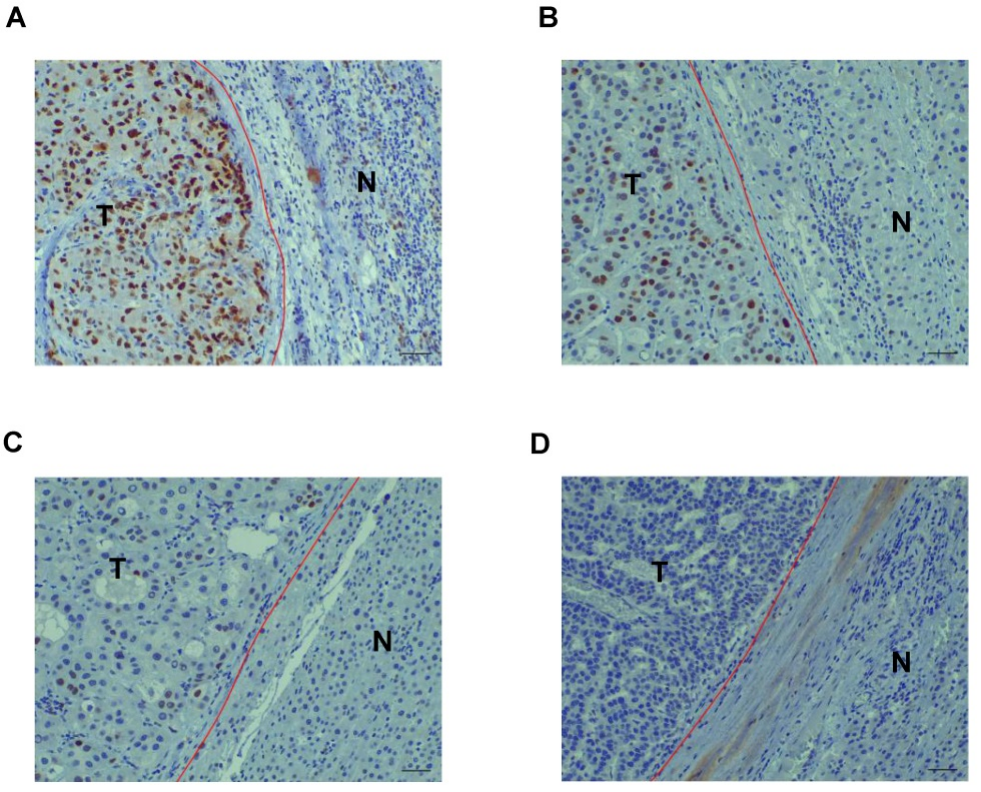
** Immunohistochemistry (IHC) staining of FIGN in hepatocellular carcinoma.** Strong (A), moderate (B), mild (C) or negative (D) nuclear expression of FIGN in carcinoma tissues. Scale bar, 10 µm. T: tumor region of HCC; N: paired adjacent normal hepatic tissue.

The correlation between FIGN expression detected by immunohistochemistry and clinicopathological characteristics of 216 patients with HCC is shown in **Table 1.** The expression of FIGN was low in 149 tumors (69.0%) and high in 67 tumors (31.0%) (**Figure 1A and B**). A remarkable positive association was observed between FIGN expression and hepatitis B surface antigen (HBs Ag) positive rate (*P* = 0.021). The proportion of tumor with incomplete capsule in high FIGN expression group was higher than that in low FIGN expression group, the difference was statistically significant (*P* = 0.036). HCC patients with high FIGN expression more frequently suffered from tumors with MVI and PVTT than individuals with low FIGN expression (*P* < 0.05 or *P* < 0.01). In addition, FIGN expression was significantly correlated with TNM stages (*P* = 0.039). However, no remarkable relationship was observed between FIGN expression and any other clinicopathological features including age at surgery, gender, tumor diameter, number of tumors, satellite nodule and BCLC stage.

### FIGN expression in HCC tissues

**Figure 2:**
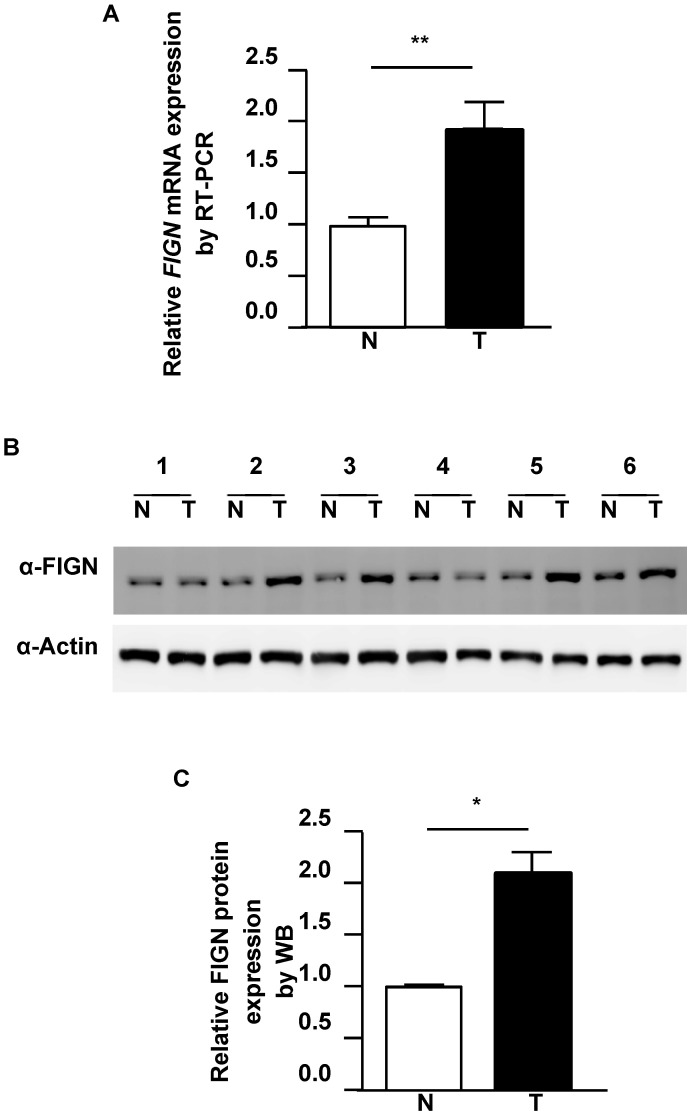
** FIGN is elevated in hepatocellular carcinoma.** (A) Representative qRT-PCR statistical results are shown (n = 24). (B) FIGN protein expression was significantly higher in tumor tissues by western blot (WB). Actin was used as the control. (C) Representative WB statistical results are shown (n = 6). Densitometric quantitation of the immunoblots was performed using Image J software. All data are shown as mean ± standard deviation. *P < 0.05, **P < 0.01, in comparison with adjacent normal tissues. T: tumor region of HCC; N: paired adjacent normal hepatic tissue.

To delineate the extent of FIGN alteration in HCC, we analyzed FIGN expression in tumor regions and paired adjacent normal liver from HCC patients. qRT-PCR data showed that the mRNA expression of *FIGN* in HCC tissues was about 1.8-fold higher than that in the paired healthy liver (*P* = 0.005, **Figure 2A**). Consistently with the RT-PCR results, the protein expression of FIGN was increased to 2.1-fold in HCC compared to adjacent healthy tissues (*P* = 0.043, **Figure 2B and C**). Taken together, FIGN is overexpressed in HCC.

### Correlation between FIGN expression and HCC prognosis

**Figure 3:**
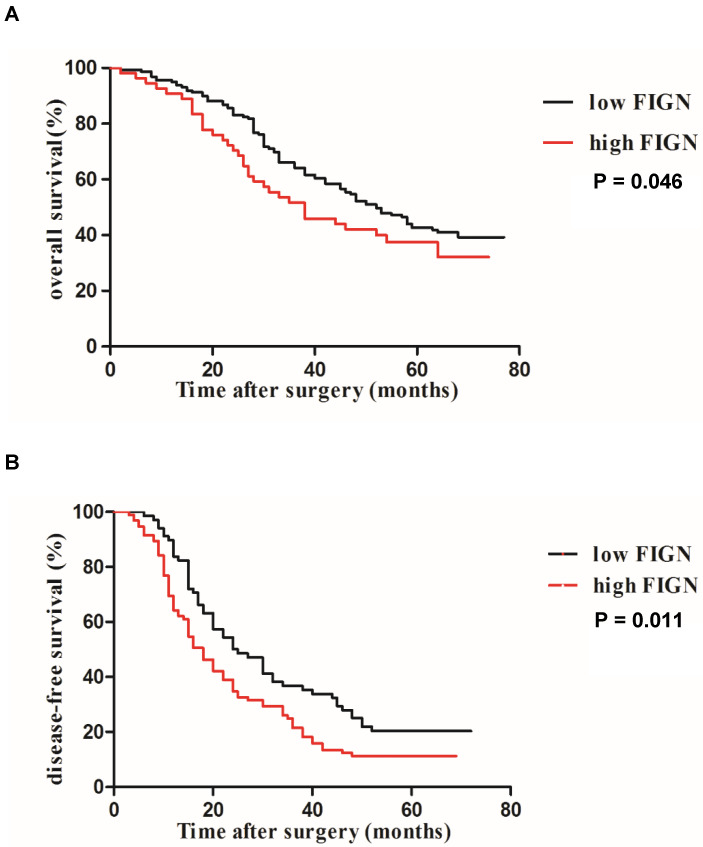
** Kaplan-Meier analysis of DFS and OS curves in HCC patients.** (A) OS curve analysis by stratified according to FIGN expression (log-rank P = 0.046); (B) DFS curve analysis by stratified according to FIGN expression (log-rank P = 0.011).

The Kaplan-Meier survival analysis for OS and DFS according to FIGN protein expression is presented in **Figure 3A and B.** Survival analyses showed that FIGN was significantly correlated to tumor progression and adverse prognosis. Patients with high FIGN expression had a significantly shorter median OS (38 months, 95 % CI: 26.2-77.5 months) than patients with low FIGN expression (52 months, 95 % CI: 23.4-66.1 months) (*P* = 0.046). When DFS was evaluated, high FIGN expression cases were easier to have inferior survival compared to the cases with low FIGN expression (*P* = 0.011).

Using the Cox proportional hazards model, we assessed the independent prognostic value of several clinicopathological characteristics and FIGN expression for DFS and OS with multivariate analyses. The results of Cox regression analyses for OS and DFS are displayed in **Table 2.** The low FIGN expression group had a significantly longer DFS (*P* = 0.001) and OS (*P* = 0.036) compared to the high FIGN expression group, indicating that the elevated FIGN could act as an independent adverse prognostic factor for DFS and OS. Furthermore, incomplete tumor envelope (*P* = 0.039), multiple tumors (*P* = 0.017), MVI (*P* = 0.001), PVTT (*P* = 0.001), later TNM stage (*P* = 0.027) and more advanced BCLC stage (*P* = 0.023) were also significantly associated with worse OS of patients with HCC. Similarly, tumor envelope (*P* = 0.011), number of tumor (*P* = 0.039), satellite nodule (*P* = 0.029), MVI (*P* = 0.004), PVTT (*P* = 0.001) and TNM stage (*P* = 0.001) were also independent prognostic factors for predicting DFS of patients with HCC.

## Discussion

Approximately 75% of liver cancer occurs in Asia, and China accounts for over 50% of the world's burden 1. In the past two decades with remarkable advantages in surgical therapies and systemic targeted treatments, the 5-year survival rate of HCC remains low 12. The HCC treatment efficacy depends on the tumor stage, patient performance status and liver function reserve. For early cases, hepatectomy is effective; whilst for advanced cases, systemic therapy including radiofrequency ablation, microwave ablation, percutaneous ethanol injection, transcatheter arterial chemoembolization and transplantation may not improve the poor prognosis 13. Extensive local invasion and distant metastasis are the major reasons of deaths in patients with HCC 14, 15. Therefore, the prognostic and predictive biomarkers of tumor migrate and progression is eagerly wanted for the improvement of HCC outcomes.

The present study in a population of 216 patients with HCC demonstrated that FIGN was correlated with advanced HCC factors, including HBV infection, incomplete tumor capsule, MVI, PVTT and later TNM stage. Moreover, survival analyses showed that FIGN was an independent prognostic predictor for DFS and OS. The survival analyses displayed that the FIGN expression was positively associated with HCC recurrence and adverse prognosis. Based on these data, we hypothesize that FIGN may be positively related to HCC development and progression. FIGN could be a potential predictive biomarker and a therapeutic target for HCC.

FIGN, a member of the AAA (ATPase associate with diverse cellular activities) family 16, is predominantly localized in the nucleus via its bipartite nuclear localization signal in the central region 7, 17, 18. In concordance with previous studies, we observed that FIGN was positively overexpressed in nucleus in human HCC tissues. To be a nuclear protein, FIGN promotes DNA synthesis and regulates mitosis 6, 7. Disturbances in any of these events could induce the accelerated cellular proliferation, which has been proposed to be an initial step in tumorigenesis. Besides, FIGN may also function outside of proliferation 14, 19. For example, severing of a regulator of MT reconfiguration has been proposed as an important component of efficient cell migration 8, 9, which is an essential step for tumor local invasion and distant metastasis 15. These data indicate FIGN involves in oncogenesis and cancer progression.

Here, we also found that both mRNA and protein expressions of FIGN were upregulated in HCC compared with the matched normal tissue. In agree with this, a recent publication has been shown that FIGN is strongly overexpressed in human and murine HCC 9. However, we found that 31% of human HCC tissues were strongly expressed with FIGN; the rate of strong FIGN expression was much lower (15%) in a previous study conducted in the United States 9. Moreover, our rate of HCC patients with HBs Ag positive was 64.4% which is almost 6-fold higher than that in the United States (10%) 20. In China, HCC are associated with cirrhosis related to chronic HBV infection in most cases 21, 22. And sustained inflammation and chronical injury can upregulate *Fign* mRNA expression 9. Thus, the more HCC cases with HBV infection could be attributable to the higher rate of FIGN strong expression in our study.

By survival analyses, we found that HCC patients with high FIGN expression were in risk of a shorter survival, suggesting FIGN is negatively associated with HCC outcome. However, Riordan *et al* 9 displayed that no significant association was detected between *FIGN* mRNA expression and survival of HCC patients in America. A series of outcomes comparison between HBV related HCC (HBV-HCC) and non-HBV non-HCV HCC (NBNC-HCC) revealed that HBV-HCC patients had significantly shorter OS and DSF 23-25; while postoperative regular antiviral therapy could prolong survival the HCC patients with baseline higher HBV DNA titter 26. Above evidence indicate that HBV infection is an independent risk for adverse outcome in HCC patients. The most important risk factors for HCC are HBV and HCV, the alcohol consumption and exposition to aflatoxin B1 12, 21. The HBV or HCV related HCC are caused by chronical viral infection; whereas, the etiology of NBNC-HCC is mostly a metabolic dysregulation 27. The tumor physiological features are quite different. HBV-related HCC is the largest proportion of patients with HCC in China 22, while alcohol consumption and nonalcoholic steatohepatitis are the leading causes of HCC in America 21. Thus, these inconsistent results are possibly due to the differences in demographic and HBV infection.

Furthermore, our results showed that FIGN was positively related with incomplete tumor capsule, MVI, PVTT and advanced TNM stage. These data indicate that FIGN may regulate hepatoma development. Recently, Wang *et al* 6 demonstrated that FIGN could promote cell proliferation by upregulating DNA synthesis via enhancing folate transmembrane transport and utilization in human embryonic kidney cell line. However, Riordan *et al* 9 found that Fign did not involve in cell growth in a mouse hepatocyte cell line (TIB-73); but Fign overexpressing exhibited altered colony morphology with collective invasion into the Matrigel, suggesting Fign might promote hepatocyte migration through extracellular matrix. In addition, researchers also reported that *Fign* knockdown could block astrocyte migration, with a 47.5% reduction contrast to the control 8. They further investigated that Fign reduced MT mass by preferentially targeting tyrosinated tubulin. MT often contributes to bend the polymers and help them form loops. Therefore, Fign may mediate labile MT lengths in cells. When *Fig*n is present, most of the labile MT keep perpendicular to the plasma membrane, and the cells are able to move responding to extracellular signals; whereas when *Fign* is absent, those labile MT extend excessively and curve parallel to the plasma membrane, thus blocking normal cellular movements 8. Given cellular DNA synthesis and movement are the essential steps for tumor progression, FIGN may involve in hepatoma development by promoting proliferation, cellular local migration and distant invasion.

We acknowledge that there are several limitations in our study. First, this was a retrospective study in a relatively short observation period, and the sample size of 216 HCC patients may not great enough. Further studies containing a greater population of HCC patients will be necessary for confirming our results. Second, this study recruited patients with heterogeneous characteristics. Therefore, it is unclear whether present findings could be applied to all HCC patients stratified by major parameters. Finally, in this study, we have not concerned the precise molecular mechanism by which FIGN may promote the progression of HCC. Notwithstanding these limitations, additional study will be necessary to confirm whether FIGN could be a prognostic biomarker for HCC. Moreover, data from the current study indicate that FIGN may act as a carcinogen and inhibition of FIGN could be an effective therapeutic target for HCC. Further researches on the signaling pathway that FIGN regulates tumorigenesis and development may discover novel therapeutic option to inhibit hepatoma cell proliferation and migration.

## Conclusions

In summary, our study displayed that Fidgetin expression is positively associated with tumor stage, tumor with incomplete capsule, microvascular invasion, and portal vein tumor thrombus; but it is negatively related to overall survival and disease-free survival. These data demonstrate that Fidgetin is correlated with tumor progression and suggest a worse prognosis in hepatocellular carcinoma. Fidgetin might serve as a potential target for therapy.

## Figures and Tables

**Table 1:** FIGN expression in relation to clinicopathological characteristics in 216 patients with HCC

	Entire group (n = 216)	FIGN expression		*P* value
		Low (n = 149)	High (n = 67)	
**Age at surgery (years) n (%)**				0.551
<60	128 (59.3%)	86 (57.7%)	42 (62.7%)	
≥60	88 (40.7%)	63 (42.3%)	25 (37.3%)	
**Gender n (%)**				0.538
Male	141 (65.3%)	95 (63.8%)	46 (68.7%)	
Female	75 (34.7%)	54 (36.2%)	21 (31.3%)	
**HBs Ag n (%)**				0.021
Positive	139 (64.4%)	88 (59.1%)	51 (76.1%)	
Negative	77 (35.6%)	61 (40.9%)	16 (23.9%)	
**Diameter (cm) n (%)**				0.079
<5	113 (52.3%)	84 (56.4%)	29 (43.3%)	
≥5	103 (47.7%)	65 (43.6%)	38 (56.7%)	
**Tumor envelope, n (%)**				0.036
Complete	127 (58.8%)	95 (63.8%)	32 (47.8%)	
Incomplete	89 (41.2%)	54 (36.2%)	35 (52.2%)	
**Number of tumor, n (%)**				0.180
Solitary	177 (81.9%)	126 (84.6%)	51 (76.1%)	
Multiple	39 (18.1%)	23 (15.4%)	16 (23.9%)	
**Satellite nodule, n (%)**				0.099
Positive	43 (19.9%)	25 (16.8%)	18 (26.9%)	
Negative	173 (80.1%)	124 (83.2%)	49 (73.1%)	
**MVI n (%)**				0.023
Positive	63 (29.2%)	36 (24.2%)	27 (40.3%)	
Negative	153 (70.8%)	113 (75.8%)	40 (59.7%)	
**PVTT n (%)**				0.003
Positive	36 (16.7%)	17 (11.4%)	19 (28.4%)	
Negative	180 (83.3%)	132 (88.6%)	48 (71.6%)	
**TNM stage n (%)**				0.039
I	148 (68.5%)	109 (73.2%)	39 (58.2%)	
II/ III	68 (31.5%)	40 (26.8%)	28 (41.8%)	
**BCLC stage n (%)**				0.214
A	143 (66.2%)	103 (69.1%)	40 (59.7%)	
B/C	73 (33.8%)	46 (30.9%)	27 (40.3%)	

HBsAg: hepatitis B surface antigen; MVI: microvascular invasion; PVTT: portal vein tumor thrombus; TNM: Tumor-Node-Metastasis; BCLC: Barcelona Clinic Liver Cancer.

**Table 2:** Multivariate analysis of overall survival and disease-free survival

Covariates	Overall survival		Disease-free survival	
	HR (95%CI)	*P* value	HR (95%CI)	*P* value
Age at surgery	0.773 (0.577-1.153)	0.147	0.836 (0.753-1.172)	0.193
Gender (male vs. female)	1.872 (0.127-2.488)	0.327	1.964 (0.123-4.327)	0.772
HBs Ag (positive vs. negative)	1.676 (1.042-5.493)	0.082	1.964 (1.219-4.271)	0.142
Tumor diameter (≥ 5 vs. < 5 cm)	1.274 (0.717-6.743)	0.228	1.741 (0.665-8.736)	0.402
Tumor envelope (incomplete vs. complete)	2.743 (1.537-8.365)	0.039	3.564 (1.737-9.475)	0.011
Number of tumor (multiple vs. solitary)	4.357 (2.157-13.249)	0.017	3.728 (1.889-10.965)	0.039
Satellite nodule (positive vs. negative)	8.742 (2.834-86.851)	0.062	9.269 (0.967-127.538)	0.029
MVI (positive vs. negative)	4.287 (0.578-32.482)	0.001	3.272 (1.078-14.542)	0.004
PVTT (positive vs. negative)	17.536 (3.964-228.733)	0.001	14.218 (2.463-148.373)	0.001
TNM stage (II/ III vs. I)	12.785 (0.964-183.778)	0.027	4.924 (0.894-42.362)	0.001
BCLC stage (B/C vs. A)	10.367 (3.674-167.758)	0.023	7.183 (1.373-846.782)	0.091
FIGN expression (high vs. low)	4.569 (1.282-65.481)	0.036	6.487 (1.685-103.463)	0.001

HR: hazard ratio; CI: confidence interval.
